# Superparamagnetic iron oxide nanoparticles promote ferroptosis of ischemic cardiomyocytes

**DOI:** 10.1111/jcmm.15722

**Published:** 2020-08-11

**Authors:** Hao Zheng, Jieyun You, Xiaobo Yao, Qizheng Lu, Wei Guo, Yunli Shen

**Affiliations:** ^1^ Department of Cardiology Shanghai East Hospital Tongji University School of Medicine Shanghai China

**Keywords:** cardiotoxicity, ferroptosis, mitochondria, superparamagnetic iron oxide nanoparticles

## 1 INTRODUCTION AND BACKGROUND

Superparamagnetic iron oxide nanoparticles (SPION) have been widely used in the diagnosis and treatment for cardiovascular diseases.^1^, ^2^, ^3^, ^4^, ^5^, ^6^ Correspondingly, the myocardial tissue safety of SPION is becoming a bottleneck to seriously restrict its clinical translation. In recent years, in vitro and in vivo experiments have confirmed that SPION‐induced oxidative stress of normal myocardium in mice, leading to myocardial cell injury, apoptosis or necrosis.^7^, ^8^, ^9^ More alarmingly, SPION applied to ischemic myocardium could accumulate in the target sites for a long time with high concentration,^5^, ^6^, ^10^ thereby probably further aggravating oxidative stress injury and cardiomyocytes death.^11^, ^12^

So far, however, the specific molecular mechanism of cardiotoxicity of SPION remains unclear. Previous studies have reported that SPION‐induced apoptosis of murine macrophage (J774) cells ^13^ and necrosis of human endothelial cells.^14^ SPION can selectively induce autophagy‐mediated cell death of human cancer cells (A549).^15^ After SPION pre‐treatment, H9C2 cardiomyocytes were exposed to acrolein or H_2_O_2_, leading to reactive oxygen species (ROS) dependent cell necrosis.^7^ Our in vitro experiment showed that SPION significantly increased oxidative stress damage to overactivate autophagy and endoplasmic reticulum stress, eventually resulting in cardiomyocyte apoptosis.^12^ Furthermore, SPION could elicit IL‐1βrelease and pyroptosis in macrophages, especially with the octapod and plate morphology.^16^ Notably, it has been recently reported that sorafenib or cisplatin assembled into nano‐devices containing SPION, which are phagocytized by tumour cells and degraded into free divalent iron to accelerate Fenton reaction, leading to the lipid peroxidation burst to promote ferroptosis of tumour cells.^17^, ^18^

Taken together, SPION can induce apoptosis, necrosis, autophagy, pyroptosis or ferroptosis in vitro and in vivo studies. The discrepancy may be attributed to distinct cell types and experiments design. It has already been well documented that the toxicity of SPION is mainly due to its degradation and release of free iron to catalyse Fenton reaction, leading to oxidative stress by a large number of ROS generation.^19^, ^20^ Then, what is the downstream molecular mechanism of SPION mediated cardiotoxicity?

Ferroptosis is a novel form of regulated cell death characterized by the iron‐dependent accumulation of lipid peroxides to lethal levels, which is morphologically, biochemically, and genetically distinct from apoptosis, necroptosis and autophagy.^21^ Recent studies found that ferroptosis is not only an important pathological mechanism in the case of circulating iron overload of hemochromatosis,^22^ but also a key molecular mechanism of cellular iron overload in doxorubicin (DOX) induced cardiomyopathy.^23^ DOX induced mitochondria iron overload by down‐regulating ABCB8,^24^ a mitochondrial protein that facilitates iron export, to elicit lipid peroxidation and mitochondria dysfunction, eventually causing cardiomyocytes ferroptosis.^23^ Mice that were subjected to 30 minutes of myocardial ischemia followed by 24 hours of reperfusion had significantly higher levels of cardiac non‐heme iron, cardiac ferritin H, ferritin L and *Ptgs2* mRNA. Both ferroptosis inhibitor Ferrostatin‐1 (Fer‐1) and iron chelator Dexrazoxane (DXZ) pre‐treatment significantly reduced I/R‐induced cardiac remodelling and fibrosis, indicating that ischemia‐reperfusion could also induce cardiomyocytes iron overload to cause ferroptosis and subsequent left ventricular remodelling.^23^ Myocardial haemorrhage is a frequent complication after successful myocardial reperfusion,^25^, ^26^ which is associated with residual myocardial iron in post‐myocardial infarction (MI) patients received reperfusion therapy.^27^ It is reasonable to infer that this iron accumulation has a potential to generate excessive ROS and trigger pathological events such as ferroptosis. A previous study also confirmed that ferroptosis is a significant type of cell death in cardiomyocytes; moreover, mechanistic target of rapamycin (mTOR) was found to play an important role in protecting cardiomyocytes against excess iron and ferroptosis by regulating ROS production.^28^ In addition, glutathione peroxidase 4 (GPX4), which protects cells from ferroptosis, was down‐regulated in the early and middle stages of MI mouse model, suggesting that ferroptosis during MI was in part due to a reduction in GPX4 protein.^29^

Even though signalling pathways of ferroptosis in cardiovascular diseases is not yet well characterized, it has been confirmed that ischemia‐reperfusion (I/R) could induce mitochondrial iron overload in cardiomyocytes rather than the increase of iron content in cytoplasm.^30^ In this study, mice treated with 2,2′‐bipyridyl (BPD), which has high membrane permeability and thus is able to access mitochondria, had demonstrated protective effects on I/R myocardium, while deferoxamine (DFO) failed to protect mice against I/R damage due to poor penetrance into mitochondria. Notably, overexpression of ABCB8 in cardiomyocytes in mice reduces mitochondrial iron and protects against I/R damage,^30^ suggesting that ABCB8 might play an important role in maintaining iron homeostasis in myocardial mitochondria and regulating ferroptosis after I/R injury. Thus, it is not difficult to speculate that SPION could aggravate mitochondrial iron load in I/R myocardium. SPION applied in ischemic myocardium could be directly degraded by cardiomyocytes,^12^ leading to severe mitochondrial iron overload.

We detected prominently mitochondrial lipid peroxidation (malondialdehyde, MDA), mitochondrial membrane potential (MMP) loss and ATP depletion at 24 hours and 4 weeks after SPION injected into the peri‐infarcted zones of myocardial ischemia‐reperfusion rats compared with the control group (all *P* < .01). We found that iron content of mitochondria was significantly higher than that in the control group (*P* < .001), and the distorted mitochondria were observed by transmission electron microscopy in the SPION group, suggesting that SPION have the potential to destroy mitochondrial structure and function by inducing mitochondria iron overload (data not published). Mitochondria are the major site of iron metabolism and ROS production, thereby cardiomyocytes iron accumulation is especially prone to induce mitochondria iron overload to trigger mitochondrial oxidative damage. Based on the above results, we speculate that SPION might further promote ferroptosis to aggravate left ventricular remodelling and cardiac deterioration by inducing severe mitochondria iron overload to promote lipid peroxidation burst.

## 2 HYPOTHESIS

**FIGURE 1:**
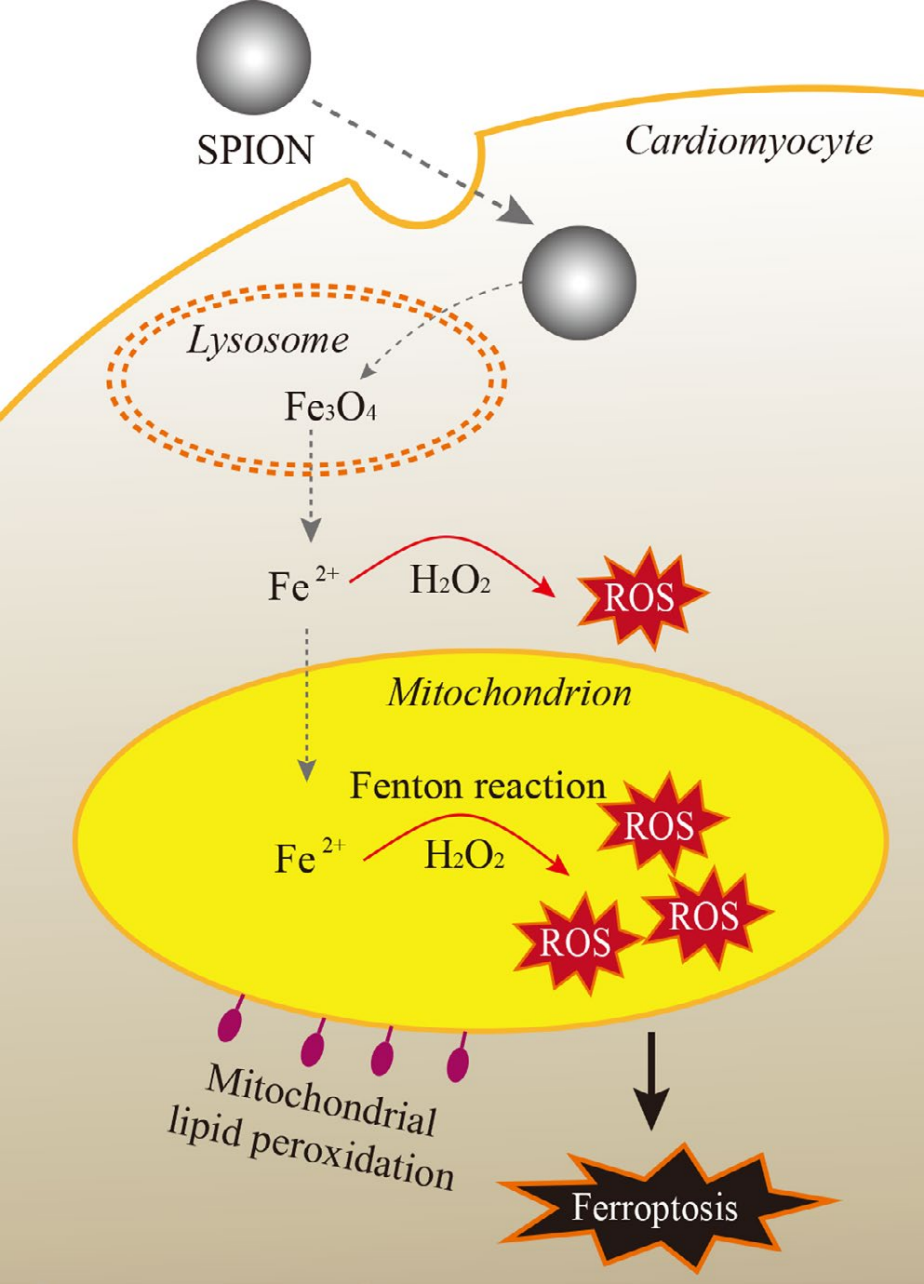
SPION were internalized into cardiomyocytes and further degraded into free ferrous iron in lysosomes. The free ferrous iron entered into mitochondria, resulting in lipid peroxidation of mitochondria to trigger ferroptosis in cardiomyocytes by a large amount of ROS produced via Fenton reaction

To summarize, we speculate that SPION applied to ischemic myocardium could exacerbate cardiomyocytes ferroptosis to worsen left ventricular negative remodelling through inducing mitochondria iron overload to catalyse sustained Fenton reaction, eliciting lipid peroxidation burst（as shown in Figure 1). This hypothesis needs to be verified by animal experiments. Firstly, the mitochondrial iron metabolism, lipid peroxidation, morphology and function of mitochondria and ferroptosis should be carefully detected after SPION injected into the peri‐infarcted zones of myocardial ischemia‐reperfusion rats. Secondly, the rat models of myocardial ischemia‐reperfusion were randomly divided into different groups to, respectively, treated with apoptosis inhibitor, necrosis inhibitor, autophagy inhibitor and ferroptosis inhibitor, in order to verify whether ferroptosis play a pivotal role in cardiomyocytes death induced by SPION. Thirdly, SPION were injected into the myocardium of I/R mice model, in which *Mlkl* ^−/−^ or *Fadd* ^−/−^ *Mlkl* ^−/−^ mice were employed to respectively block the pathways of myocardial cell necroptosis or apoptosis, in order to further illustrate whether ferroptosis is the main pathway of SPION‐induced cardiomyocytes death. Fourthly, developing SPION modified by mitochondrial iron chelator or mitochondrial‐targeted antioxidant, the effects of two strategies to improve the myocardial safety of SPION should be comprehensively investigated in vitro and in vivo experiments, potentially promoting the clinical transformation of SPION in cardiovascular field.

## 3 IMPLICATION

The cardiotoxicity of SPION limits its diagnostic or therapeutic application in the cardiovascular field. It is helpful to promote the clinical transformation of SPION in cardiovascular field through rescuing the key target of SPION‐induced cardiomyocyte ferroptosis to improve the myocardial tissue safety. If our hypothesis is true, given that SPION mainly induce mitochondria iron overload of ischemic cardiomyocytes to catalyse lipid peroxidation and exacerbate ferroptosis, and then it is expected to significantly inhibit ferroptosis induced negative remodelling of ischemic myocardium by mitochondrial iron chelator or mitochondrial‐targeted antioxidant peptide modifying SPION to effectively protect mitochondria.

## CONFLICT OF INTEREST

The authors confirm that there are no conflicts of interest.
